# The ArchBridge™ Digitally Integrated Workflow: A Fully Digital, Model-Free Workflow for Implant-Supported Overdenture Rehabilitation

**DOI:** 10.7759/cureus.115310

**Published:** 2026-08-27

**Authors:** Joc Jernigan, Gregori M Kurtzman

**Affiliations:** 1 Dentistry, Denture Care and Implant Solutions, Raleigh, USA; 2 General Dentistry and Implantology, Private Practice, Silver Spring, USA

**Keywords:** archbridge, digital dentistry, digital workflow, implant-supported overdenture, intraoral scanning

## Abstract

Implant-supported overdentures provide predictable improvements in retention, stability, function, and patient satisfaction compared with conventional complete dentures. However, conventional prosthetic workflows may require multiple impressions, casts, jaw relation records, and laboratory procedures. Although digital dentistry has simplified many restorative procedures, complete-arch implant scanning remains challenging because edentulous arches provide limited stable landmarks for image stitching.

This article describes the clinical application of the ArchBridge™ digitally integrated workflow (ROE Dental Laboratory, Independence, Ohio) system as a fully digital, model-free workflow for fabrication of an implant-supported overdenture that can be utilized as a removable or fixed prosthesis. Following placement of definitive LOCATOR® (Zest Dental Solutions, Carlsbad, CA) abutments, uniquely identifiable ArchBridge™ scan bodies were used to digitally register implant position and orientation. The existing overdenture was relined with light-body polyvinyl siloxane and scanned extraorally, and scans of the opposing arch and interocclusal relationship completed the digital record. These datasets were merged to preserve the patient's established tooth arrangement, vertical dimension of occlusion, occlusal relationships, esthetics, and soft tissue contours during design of the definitive prosthesis. The definitive overdenture was fabricated digitally, with the retentive housings incorporated at the laboratory using a proprietary patent-pending processing technique that eliminates the need for poured models and chairside housing pickup.

The ArchBridge™ workflow integrates implant registration and the patient's previously validated prosthetic information into a comprehensive digital dataset. This approach reduces reliance on conventional analog procedures while allowing the laboratory to fabricate the prosthesis and process the attachment housings directly from digital records. An additional advantage is the creation of a permanent digital archive that may facilitate subsequent repair, duplication, replacement, relining, or conversion to a fixed implant-supported restoration without repeating many conventional clinical procedures.

The ArchBridge™ workflow provides a completely digital, model-free approach to implant-supported overdenture rehabilitation. By combining digital implant registration, preservation of an existing clinically validated prosthesis, and laboratory processing of the attachment housings from digital records, the technique can simplify clinical and laboratory procedures while maintaining established prosthetic parameters. Further clinical studies are needed to quantitatively evaluate accuracy, treatment efficiency, patient outcomes, and long-term prosthetic performance.

## Introduction

Implant-supported overdentures have become a predictable treatment option for the rehabilitation of edentulous patients, providing significantly greater retention, stability, masticatory efficiency, patient satisfaction, and oral health-related quality of life than conventional complete dentures [1-4]. Advances in implant design, surface technology, digital treatment planning, and CAD/CAM manufacturing have further improved the predictability of implant overdenture therapy while enhancing prosthetic precision and reducing laboratory variability [5-7].

Despite these advances, accurately transferring implant position while preserving the patient's established occlusion, vertical dimension of occlusion (VDO), tooth arrangement, facial support, phonetics, and soft tissue contours remains one of the greatest challenges in full-arch implant prosthodontics. Conventional implant-level impressions using splinted impression copings and elastomeric materials continue to provide excellent accuracy but are technique sensitive, require multiple laboratory procedures, and are susceptible to errors introduced during impression making, cast fabrication, and analog processing [8-10].

Digital dentistry has simplified many restorative procedures by allowing implant position, soft tissue morphology, and occlusal relationships to be captured digitally. Although intraoral scanners demonstrate excellent accuracy for partially edentulous arches, complete-arch implant scanning remains more challenging because edentulous ridges provide few stable landmarks for image stitching over long distances [11-14]. As a result, several digital workflows have been introduced to improve full-arch accuracy while simplifying communication between the clinician and laboratory. Recent investigations have demonstrated that direct intraoral digital scans can provide dimensional accuracy and reliability comparable to extraoral digitization of poured dental models, further supporting the incorporation of direct digital acquisition into contemporary restorative workflows [15].

Implant-supported prostheses utilizing LOCATOR® (Zest Dental Solutions, Carlsbad, CA) attachments may be designed as either patient-removable overdentures or, with the LOCATOR FIXED® (Zest Dental Solutions, Carlsbad, CA) attachment system, as restorations that remain fixed for the patient and are removable only by the clinician. Although both approaches utilize implant attachments for prosthetic retention, they differ in how the definitive prosthesis is managed by the patient. The ArchBridge™ workflow (ROE Dental Laboratory, Independence, Ohio) can be incorporated into either approach. The clinical case presented in this report utilized LOCATOR FIXED® attachments and therefore represents the fixed, clinician-removable application of the workflow.

The principal digital acquisition and registration steps of the ArchBridge™ workflow are similar whether conventional removable LOCATOR® or LOCATOR FIXED® attachments are selected. The primary difference occurs at the definitive prosthetic stage, where the attachment components incorporated into the prosthesis determine whether the restoration is patient-removable or fixed for the patient and removable only by the clinician. Thus, attachment selection modifies the retention and clinical management of the definitive prosthesis without substantially changing the digital scanning and registration sequence described in this report.

## Technical report

**Figure 1:**
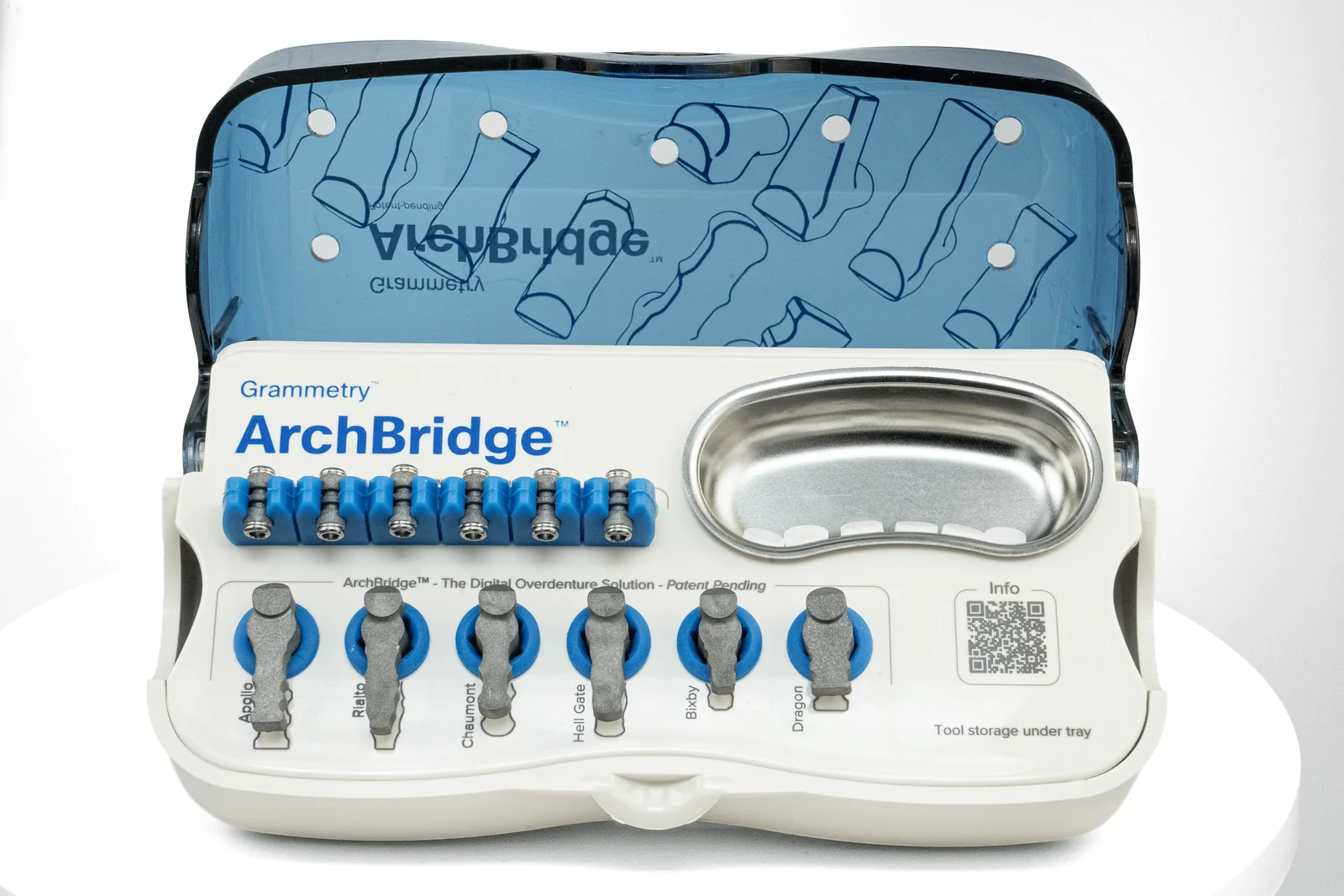
The ArchBridge™ for overdenture full-arch scan body system kit The ArchBridge™ kit (ROE Dental Laboratory, Independence, Ohio) includes uniquely identifiable scan bodies and double-sided overdenture scan analogs, used to accurately capture implant position and orientation during the digital overdenture workflow.

The ArchBridge™ for Overdenture digital workflow (ROE Dental Laboratory, Independence, Ohio) addresses these limitations by combining uniquely identifiable scan bodies with digital capture of the patient's existing denture or overdenture (Figure 1). Rather than functioning solely as an impression technique, the workflow creates a comprehensive digital record that integrates implant position, soft tissue anatomy, occlusion, vertical dimension of occlusion (VDO), and the established prosthetic contours into a single virtual dataset. By using the patient's existing prosthesis as a restorative template, many of the clinical records traditionally obtained during overdenture fabrication can be preserved digitally and transferred directly to the definitive restoration, including a model-free workflow to process the overdenture housings at the lab utilizing a proprietary patent-pending technique.

An important advantage of this approach is the creation of a permanent digital archive. Should the overdenture require repair, duplication, replacement, or future conversion to a fixed implant prosthesis, the original digital records can be retrieved without repeating many conventional impression and jaw relation procedures. This has the potential to reduce chair time, simplify laboratory procedures, and improve long-term maintenance of implant-supported prostheses.

This article describes the clinical application of the ArchBridge™ for Overdenture digital workflow for implant-supported overdentures, outlines the clinical and laboratory procedures, and discusses how digital preservation of implant and prosthetic information can improve treatment efficiency, restorative accuracy, and long-term maintenance.

Clinical significance

The ArchBridge™ for Overdenture workflow eliminates the need for conventional implant impressions by combining uniquely identifiable scan bodies with digital capture of the patient's existing denture or overdenture. In a single digital workflow, implant position, soft tissue contours, occlusion, VDO, and established prosthetic tooth arrangement are preserved and incorporated into the definitive restoration.

Rather than recreating records that have already been clinically validated, the workflow uses the existing prosthesis as a restorative template, allowing proven esthetics, phonetics, facial support, and occlusal relationships to be transferred to the definitive prosthesis. This approach reduces clinical appointments, minimizes laboratory procedures by utilizing a proprietary in-lab processing technique, and streamlines communication between the clinician and laboratory.

An equally important advantage is the creation of a permanent digital patient record. Because implant position and prosthetic information are archived electronically, future repairs, relines, replacement overdentures, duplicate prostheses, or conversion to a fixed implant restoration can often be accomplished with substantially fewer clinical procedures. This improves long-term maintenance while reducing chair time for both the clinician and the patient. For implant-supported overdenture therapy, the ArchBridge™ for Overdenture workflow represents a practical digital solution that improves restorative efficiency while maintaining the accuracy and predictability required for long-term clinical success.

The ArchBridge™ for overdenture technique for digital implant overdenture prosthetics

Digital workflows continue to improve implant prosthodontics by simplifying communication between the clinician and laboratory while reducing the number of clinical and laboratory procedures required to fabricate implant-supported restorations. Although intraoral scanning is now routine for many restorative procedures, accurately capturing multiple implants across a completely edentulous arch remains challenging because of the limited anatomic landmarks available for image stitching [11-16].

The ArchBridge™ for Overdenture workflow was developed to address these limitations by combining uniquely identifiable scan bodies, the digital capture of the patient's existing prosthesis and a proprietary patent-pending processing technique for a completely model-free experience. Unlike conventional scan bodies that rely primarily on surface geometry, the ArchBridge™ scan bodies create a distinctive three-dimensional reference pattern that improves digital registration of implant position, angulation, and rotational orientation across the arch while reducing the potential for stitching errors.

One of the greatest advantages of the ArchBridge™ for Overdenture workflow is that it is entirely digital, eliminating the need for traditional analog records and chairside housing pickup. Through the laboratory's proprietary digital processing protocol, the retentive housings are accurately incorporated into the prosthesis without requiring poured models or analog transfer procedures. While many clinicians have historically processed the housings chairside to avoid the delays and expense associated with multiple laboratory shipments, this approach can be technique-sensitive, increase chair time, and carry the risk of inadvertently locking the overdenture onto the attachments when blockout is inadequate. By eliminating these chairside procedures, the fully digital ArchBridge™ workflow provides the clinician with a definitive prosthesis that is delivered with the housings already precisely processed, reducing clinical time, simplifying delivery, and improving workflow efficiency while minimizing the potential for procedural complications [17].

Following LOCATOR® abutment placement, the ArchBridge^TM^ scan bodies are attached to each implant and an intraoral scan records implant and abutment positions together with the surrounding soft tissues. The existing overdenture is relieved as necessary to ensure passive seating, and a light-body polyvinyl siloxane (PVS) material is placed within the denture's intaglio surface to accurately record the tissue contours. The denture is then scanned extra-orally, capturing both the polished and intaglio surfaces. Digital scans of the opposing arch and the inter-occlusal relationship complete the virtual patient record.

The laboratory merges these datasets into a single digital model that reproduces implant attachment positions, soft tissue architecture, occlusion, VDO, and prosthetic contours. Using this information, the definitive overdenture can be designed with CAD/CAM technology while preserving the esthetics and occlusion that have already been clinically verified. Because much of the restorative information has already been captured, the need for additional jaw relation records and esthetic try-in appointments may be reduced or eliminated.

From a clinical perspective, the greatest advantage of the ArchBridge™ workflow is that it is completely digital from the clinician's office to the laboratory. The clinician no longer needs to obtain analog impressions, poured models, or perform chairside housing pickup procedures. Utilizing a patent-pending proprietary processing technique, the laboratory can accurately process the retentive housings directly from digital records in a completely model-free workflow. This approach may streamline treatment, reduce clinical chair time and technique-sensitive analog procedures, and simplify communication between the practice and laboratory.

A closely related advantage is the creation of a permanent digital archive. Implant positions, prosthetic design, and restorative records can be stored indefinitely and retrieved if repair, duplication, replacement, or conversion to a fixed implant-supported restoration becomes necessary. Because the complete digital dataset is preserved, many conventional impression and jaw relation procedures can often be avoided, simplifying long-term maintenance while improving efficiency for both the clinician and the laboratory.

As digital implant dentistry continues to evolve, workflows that preserve both implant and prosthetic information will become increasingly valuable. By combining accurate implant registration with digital preservation of the patient's established prosthesis, the ArchBridge™for Overdenture workflow provides a practical and efficient approach to implant-supported overdenture therapy while maintaining the restorative accuracy required for predictable long-term outcomes.

Clinical technique

Treatment begins following successful osseointegration or, in immediate-load cases, after placement of the definitive overdenture abutments. Before initiating the digital workflow, the existing prosthesis is evaluated to verify esthetics, VDO, centric occlusion, phonetics, and lip support. Because these parameters have already been clinically validated, they will serve as the foundation for the definitive prosthesis.

**Figure 2:**
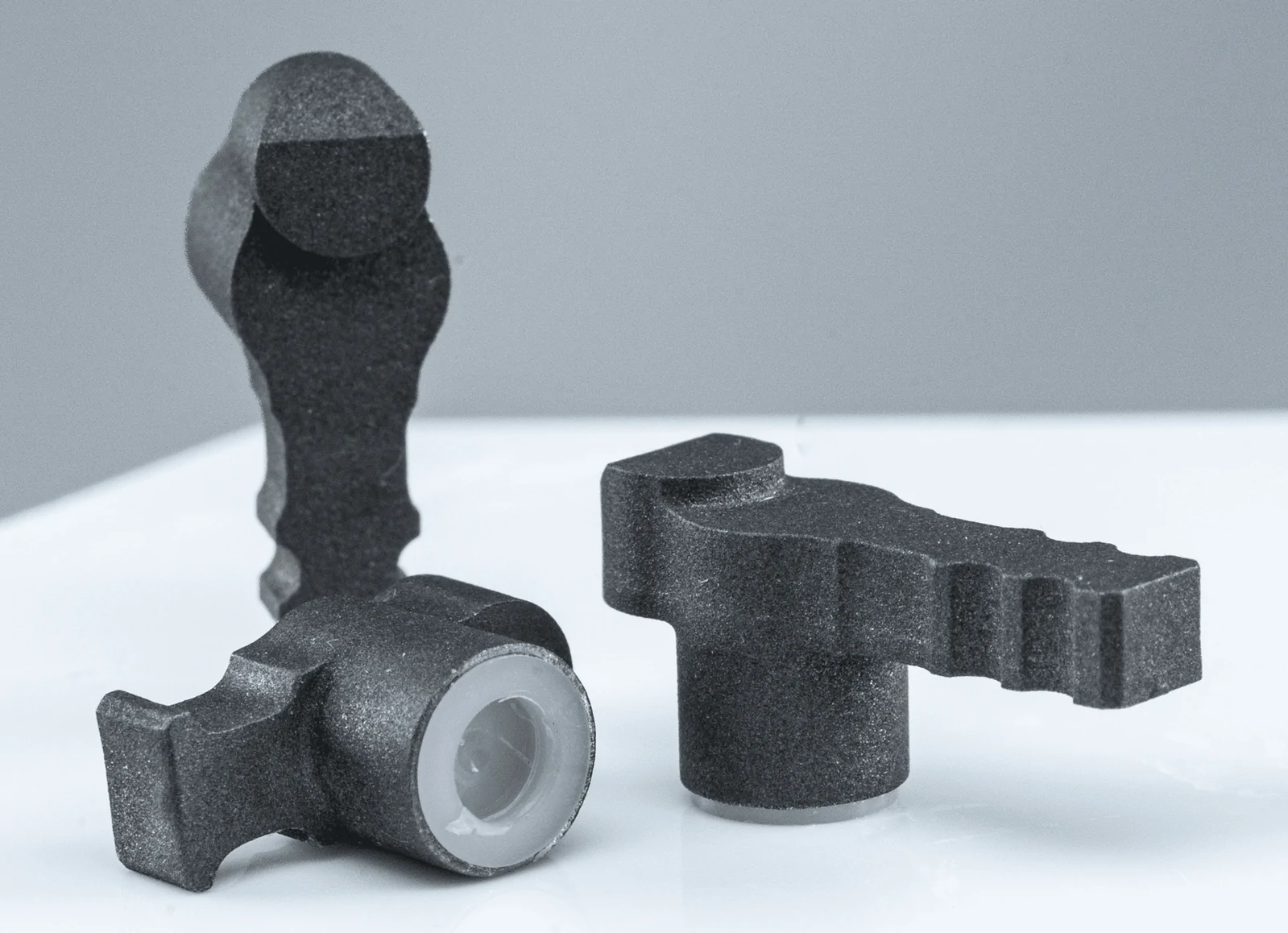
The ArchBridge™ for LOCATOR® Overdenture scan bodies ArchBridge™ bodies (ROE Dental Laboratory, Independence, Ohio) are designed to attach intraorally to the LOCATOR® abutments, providing stable seating and accurate digital registration of implant position and orientation during intraoral scanning.

After removal of the prosthesis, the healing abutments are removed, and the definitive overdenture abutments are torqued according to the manufacturer's recommendations. ArchBridge™ scan bodies are attached to each overdenture abutment intraorally. The scan bodies are specifically designed to engage the LOCATOR® attachment intraorally, providing stable seating and accurate positioning throughout the digital scanning procedure (Figure 2). An intraoral scan is then performed to capture implant position, angulation, rotational orientation, and the surrounding soft tissue anatomy. The unique geometry of the scan bodies provides stable reference points that improve registration accuracy across the edentulous arch.

Following completion of the implant scan, the scan bodies are removed. The existing denture is relieved as needed to ensure passive seating over the LOCATOR® abutments. A light-body polyvinyl siloxane (PVS) material is injected into the intaglio surface of the prosthesis, which is then seated until the material polymerizes. This records the functional soft tissue contours while preserving the established occlusal relationship.

**Figure 3:**
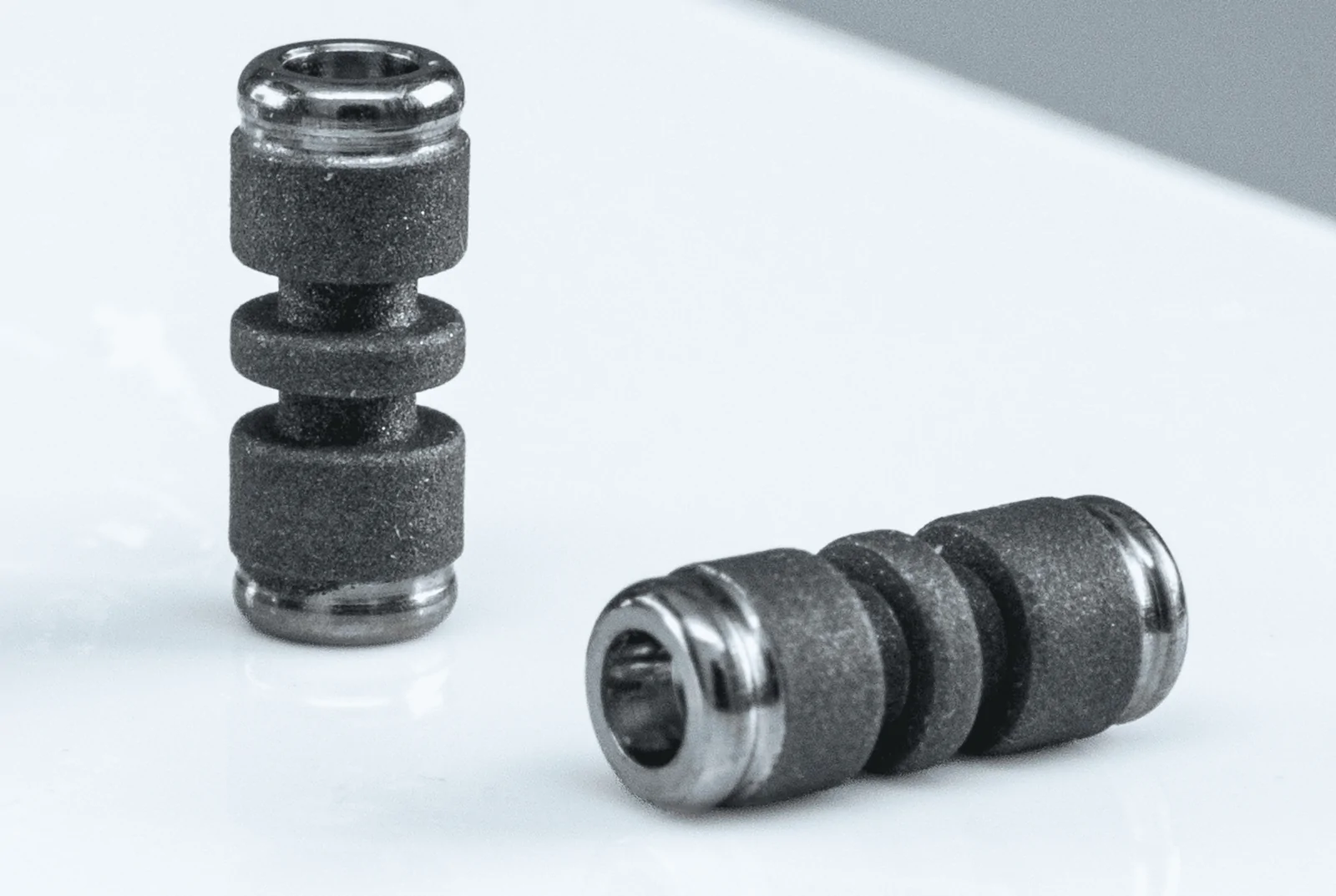
ArchBridge™ double-sided scan LOCATOR® analogs These ArchBridge™ (ROE Dental Laboratory, Independence, Ohio) double-sided scan LOCATOR® analogs are used during 360-degree extraoral scanning of the existing prosthesis to aid in recording the attachment positions within the prosthetic scan.

The denture is removed and scanned extraorally to capture both the polished and intaglio surfaces. ArchBridge™ double-sided scan analogs are used during the 360-degree extraoral scanning of the existing prosthesis to aid in recording the attachment positions within the prosthetic scan (Figure 3). Digital scans of the opposing arch and an interocclusal record complete the virtual patient record. Direct digital registration of interocclusal relationships has demonstrated accuracy in recording occlusal contacts compared with conventional articulating media in patients with natural dentition, supporting its use for virtual articulation within digital restorative workflows.

These files are transmitted electronically to the laboratory, where the intraoral implant scan, extraoral prosthesis scan, opposing arch, and interocclusal record are incorporated into a single digital dataset to design the definitive prosthesis. Using this information, the laboratory designs the definitive prosthesis while maintaining the patient’s established tooth arrangement, VDO, occlusion, and esthetics. Detailed aspects of the proprietary registration and laboratory processing protocol were not available to the authors, and the accuracy of the merged datasets was not independently quantified in this clinical report. When a milled implant bar is prescribed, a computer-aided design and computer-aided manufacturing (CAD/CAM) verification jig may be fabricated to confirm passive intraoral fit before fabrication of the definitive framework [17,18]. At the delivery appointment, the definitive overdenture is then inserted and evaluated for retention, stability, occlusion, esthetics, phonetics, and oral hygiene access. Minor occlusal adjustments are completed as needed before the patient is instructed in prosthesis insertion, removal, and long-term maintenance.

Case example

**Figure 4:**
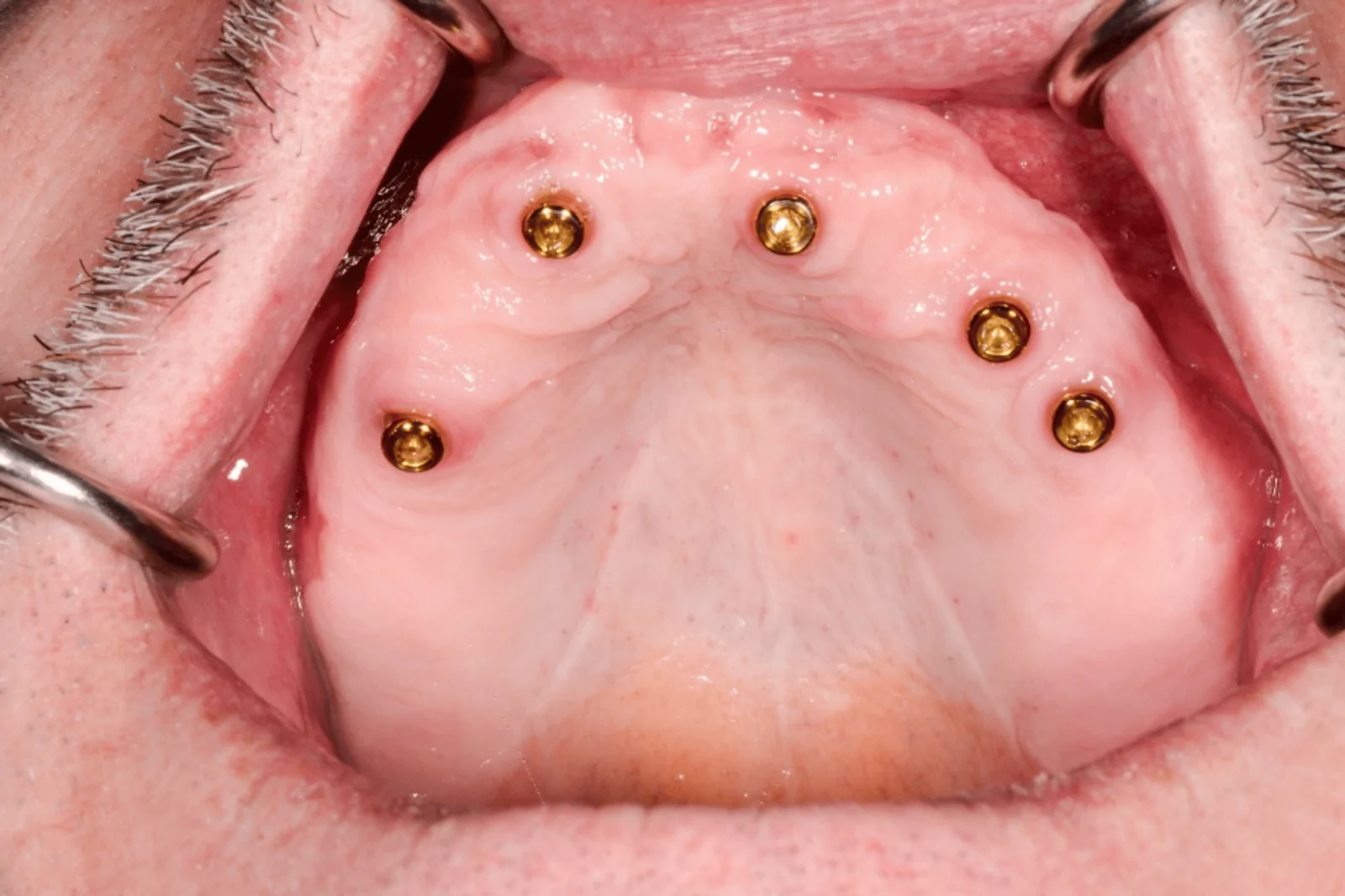
LOCATOR® abutments placed intraorally on the implants in a patient LOCATOR® (Zest Dental Solutions, Carlsbad, CA)

**Figure 5:**
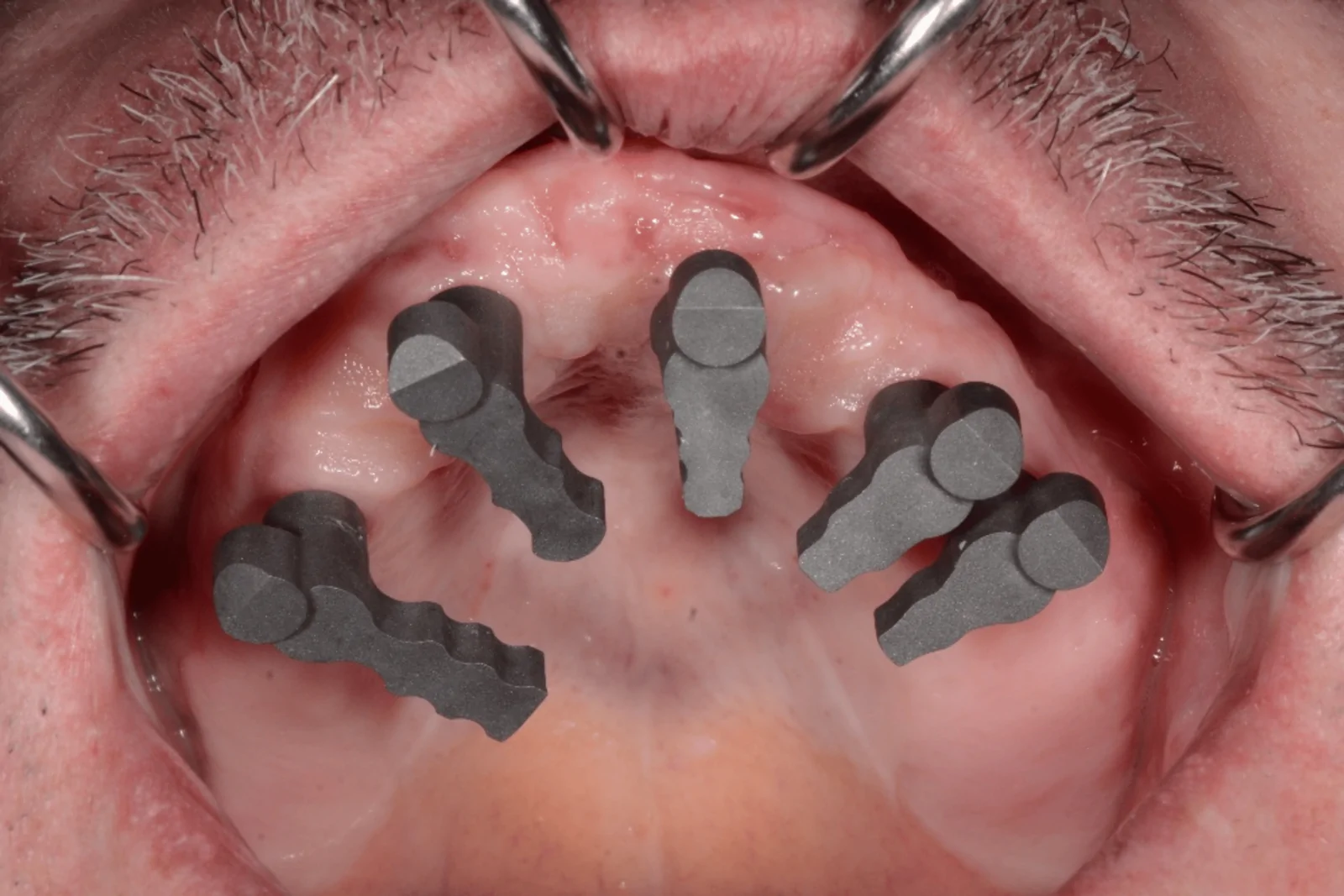
ArchBridge™ scan bodies placed onto the LOCATOR® abutments intraorally in preparation for scanning ArchBridge™ (ROE Dental Laboratory, Independence, Ohio); LOCATOR® (Zest Dental Solutions, Carlsbad, CA).

**Figure 6:**
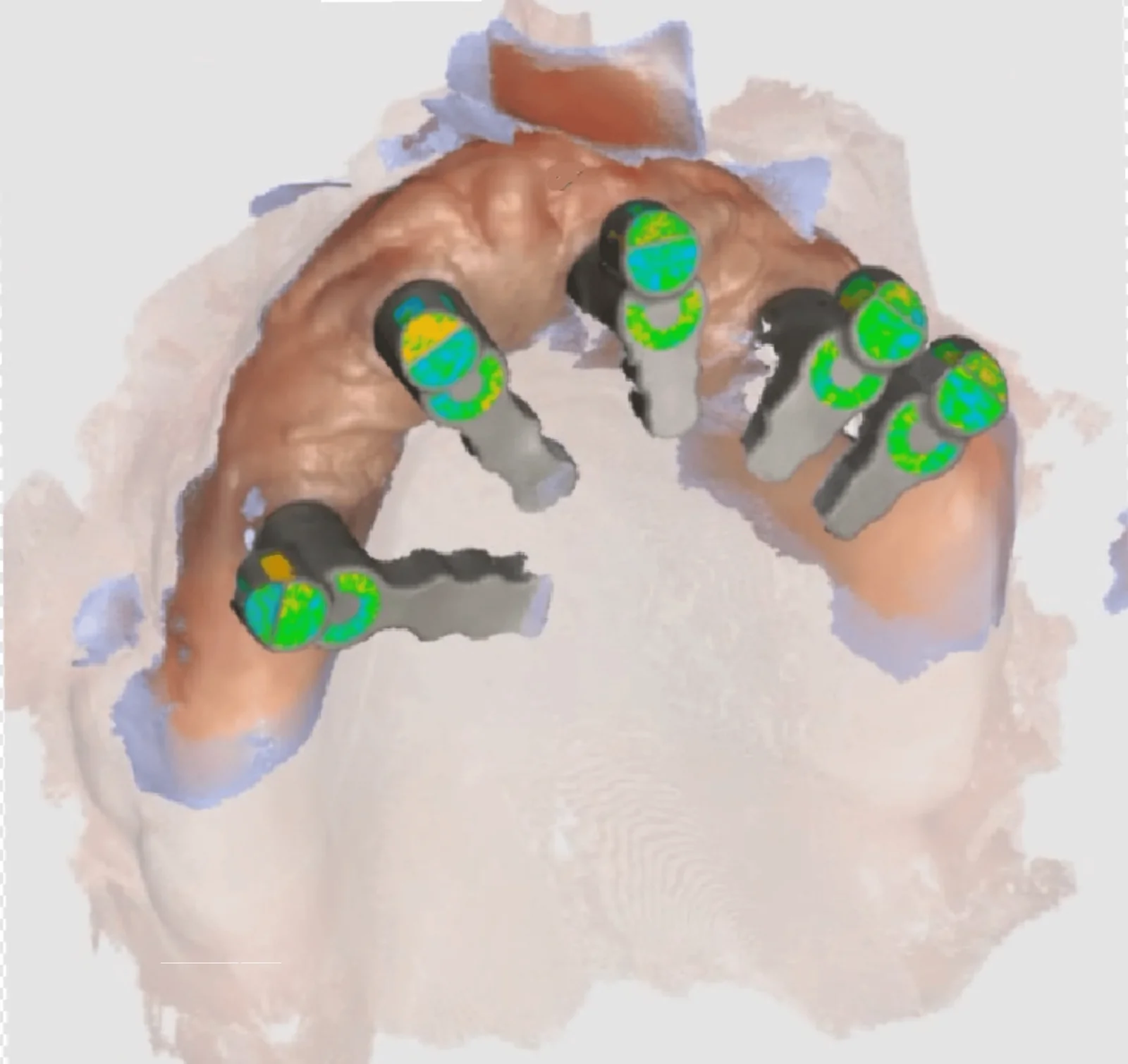
Virtual image of the ArchBridge™ scan body position recognition in the Medit SmartX software ArchBridge™ (ROE Dental Laboratory, Independence, Ohio); Medit SmartX (Medit Corp., Yeongdeungpo-gu, Seoul).

After implant osseointegration, LOCATOR® abutments were placed on the implants following removal of the healing abutments (Figure 4). ArchBridge™ scan bodies were then inserted onto the LOCATOR® abutments, allowing the intraoral scanner to accurately capture the three-dimensional implant positions (Figure 5). The resulting digital scan generated a virtual model of the maxillary arch with the scan bodies registered to their respective implant locations, creating the foundation for the prosthetic design (Figure 6).

**Figure 7:**
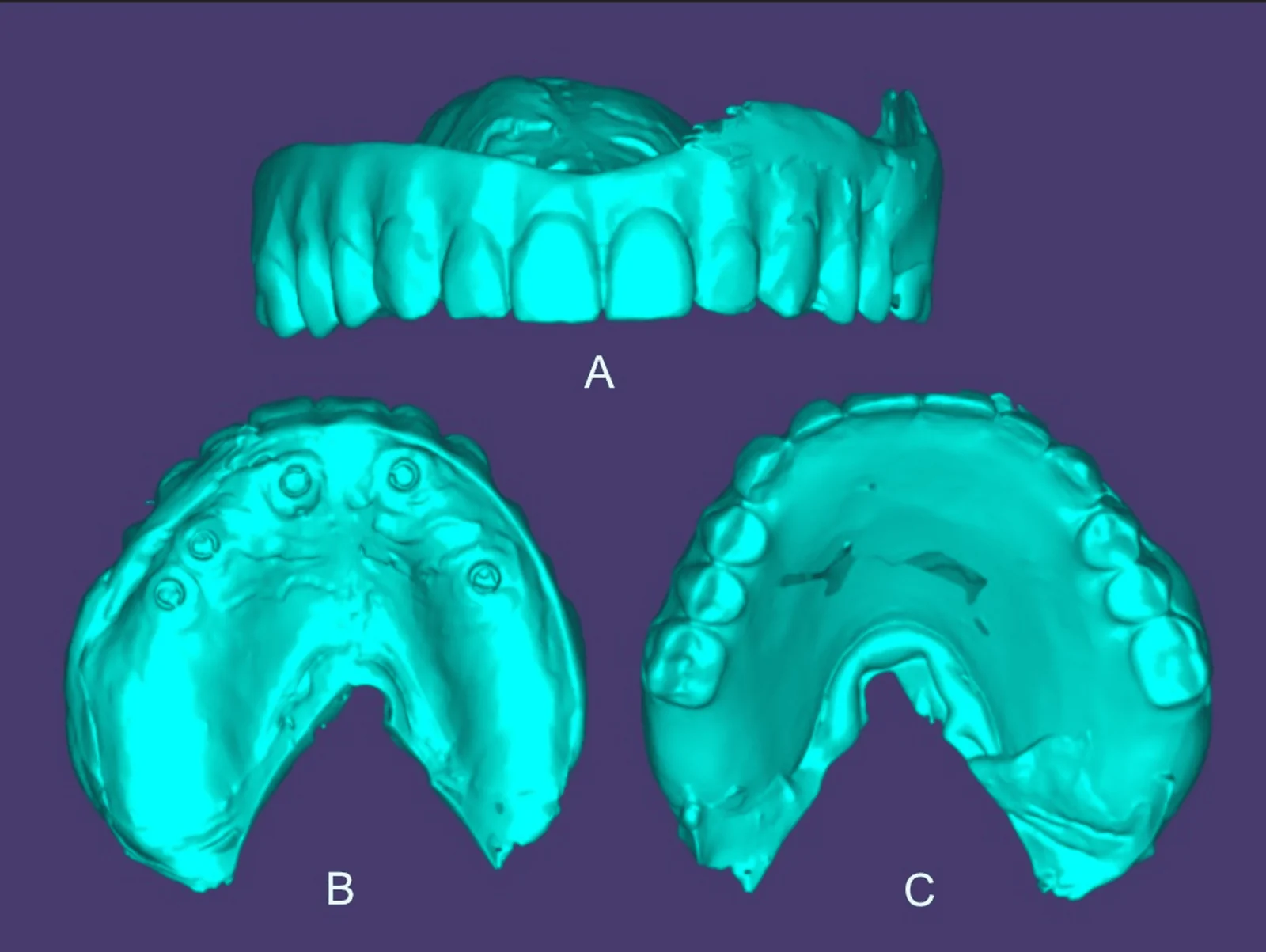
Scan of the relined maxillary denture Maxillary denture shown from facial (A), intaglio (B), and occlusal views (C).

The existing denture was relined with light-body VPS (Genie®, Sultan Healthcare) over the LOCATOR® abutments and then scanned extra-orally to capture orientation of the arch anatomy with the LOCATOR® abutments while preserving the patient's established tooth arrangement, VDO, and occlusal relationship with the mandibular arch (Figure 7). The scan files were then uploaded to the lab (ROE Dental Lab).

**Figure 8:**
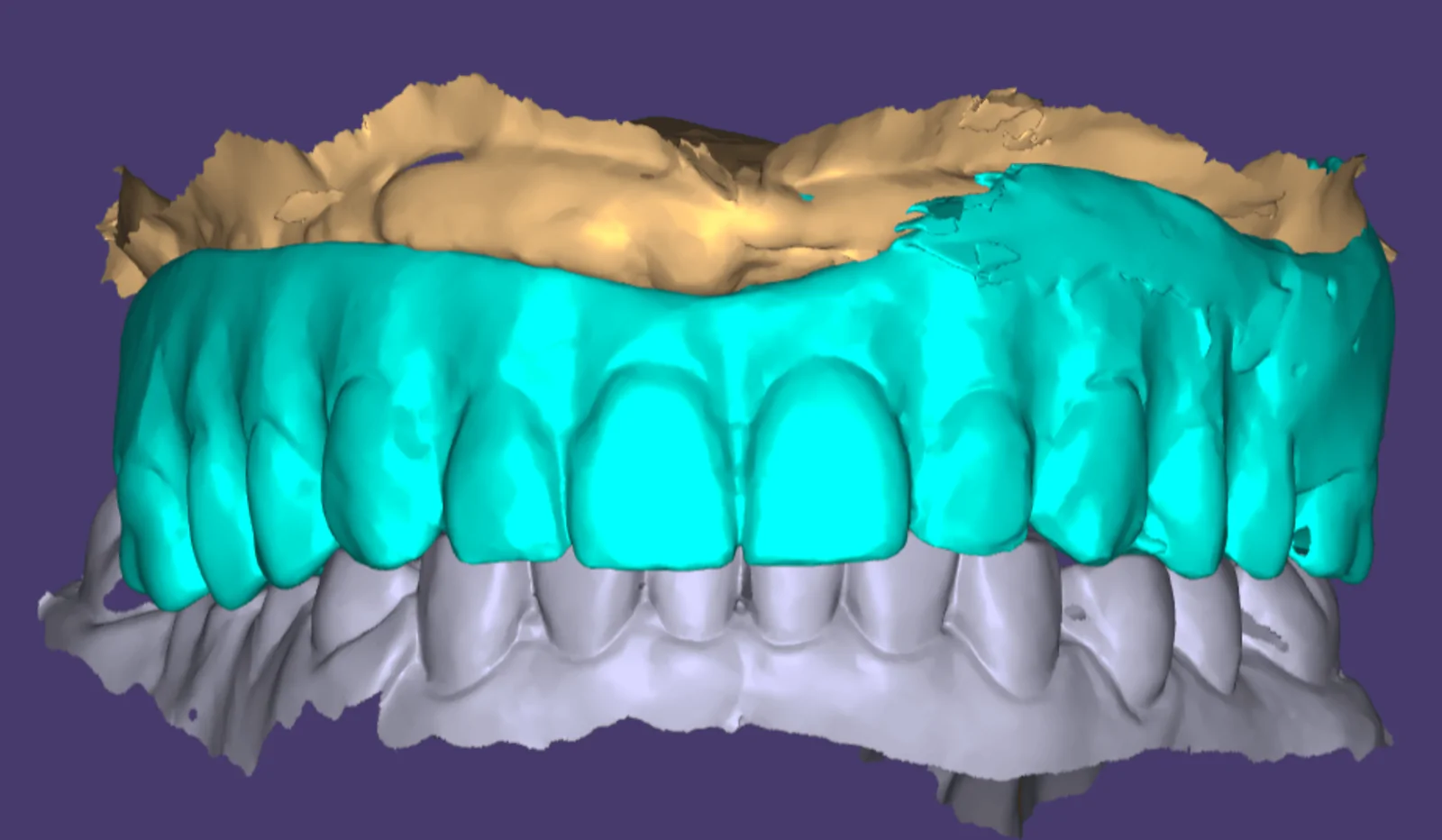
Virtual facial view of the scanned relined denture articulated with the lower arch

**Figure 9:**
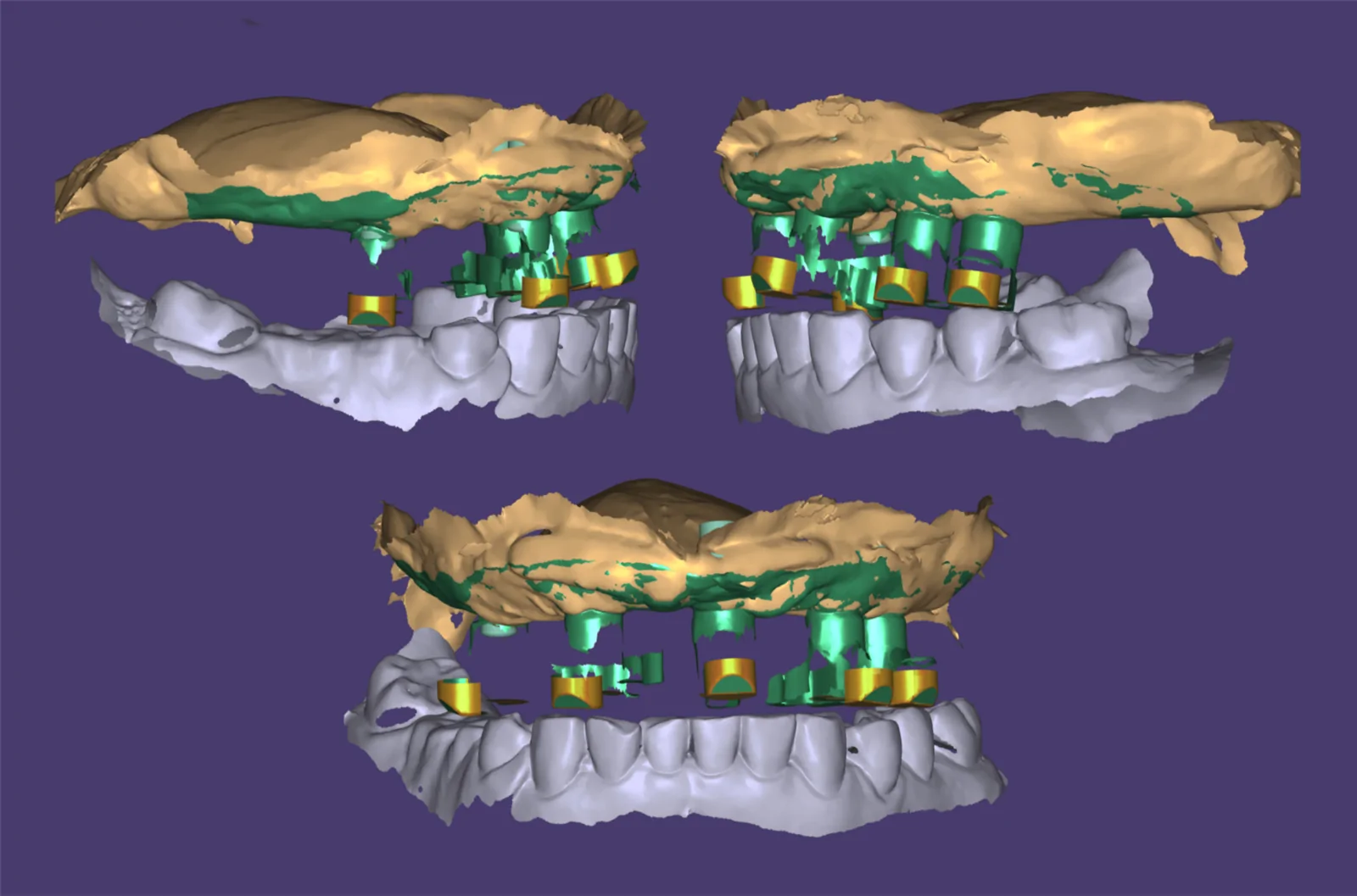
The virtual models with the scan bodies on the LOCATOR® attachments following articulation using the virtual scan of the denture LOCATOR FIXED® (Zest Dental Solutions, Carlsbad, CA).

The overdenture scan was then articulated with the opposing arch at the lab, allowing the patient's existing esthetics and occlusion to be transferred directly into the digital workflow (Figure 8). Using the datasets, the laboratory superimposed the overdenture scan onto the virtual implant model, accurately relating the prosthetic tooth arrangement to the implant positions recorded by the scan bodies (Figure 9).

**Figure 10:**
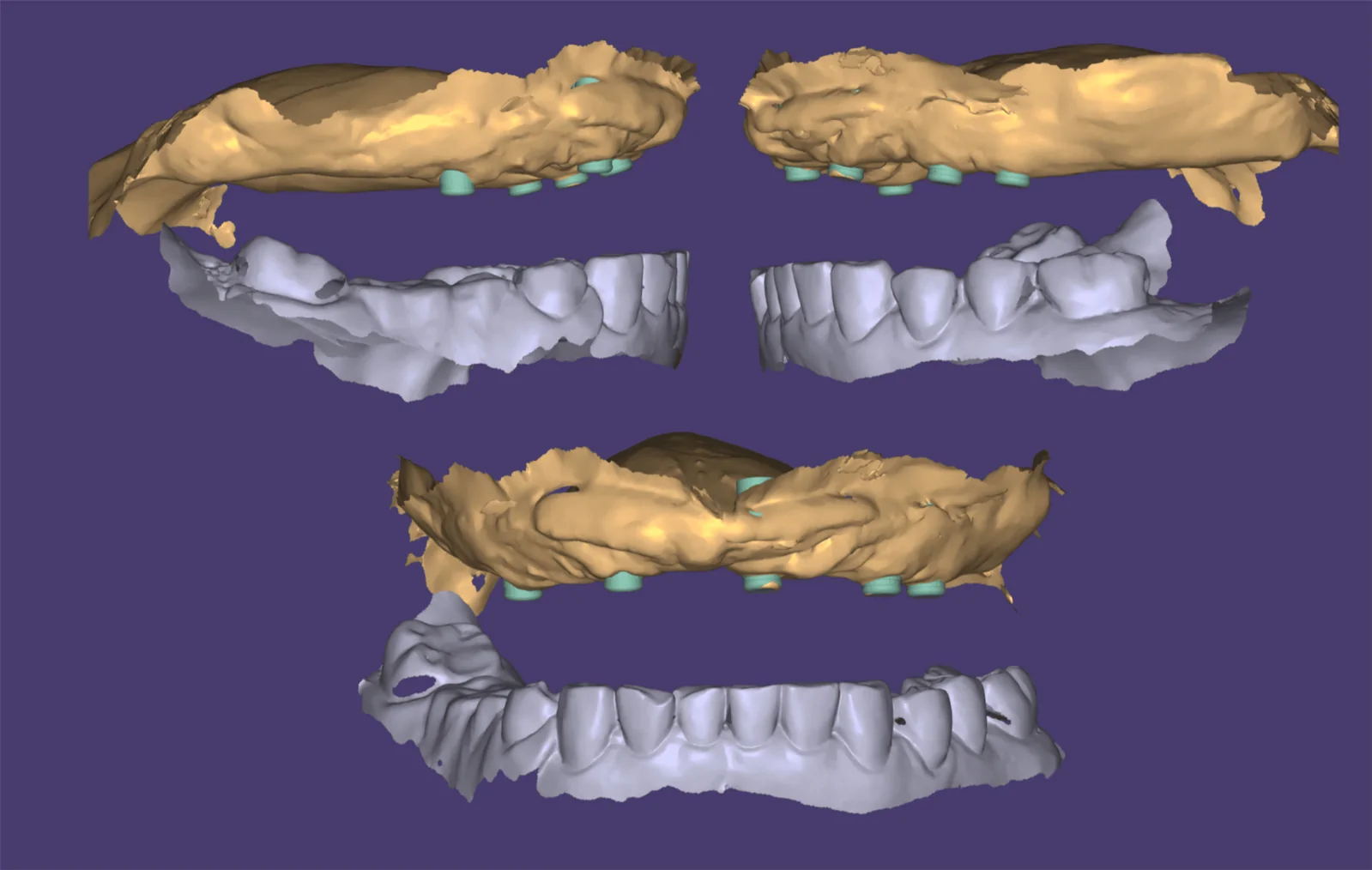
The articulated virtual models of the dentures These models demonstrate the available space for the overdenture based on the scanned denture.

**Figure 11:**
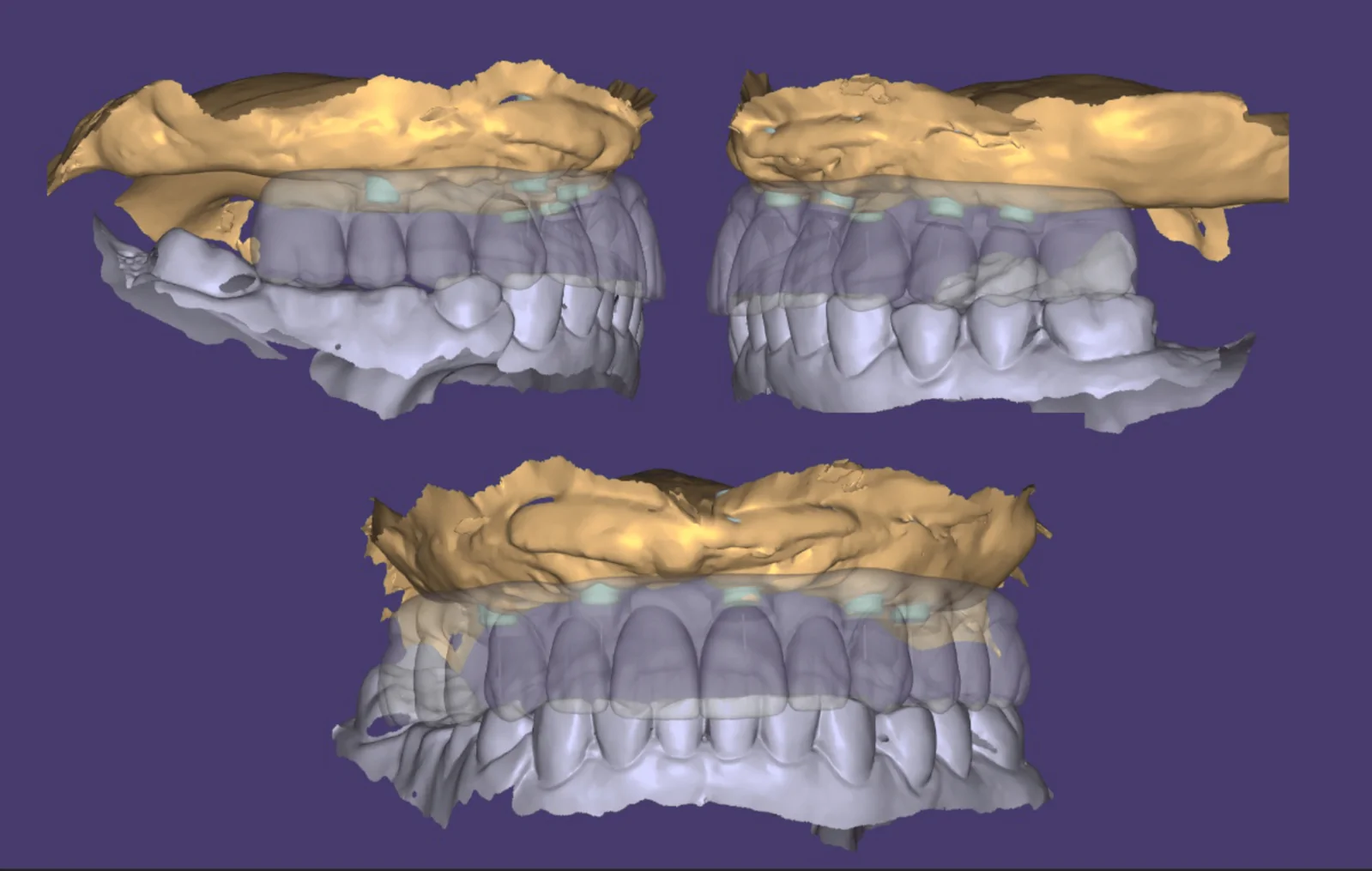
The virtual designed overdenture related to the LOCATOR® attachments articulated with the lower arch from various views LOCATOR FIXED® (Zest Dental Solutions, Carlsbad, CA).

Once the datasets were merged, the software permitted evaluation of the available restorative space between the opposing dentition and the implant attachments, facilitating design of the definitive prosthesis while maintaining the patient's established occlusal scheme (Figure 10). The definitive overdenture is then designed digitally around the LOCATOR® attachments, preserving the existing tooth arrangement while optimizing the intaglio surface for placement of the LOCATOR® Fixed housings (Figure 11).

**Figure 12:**
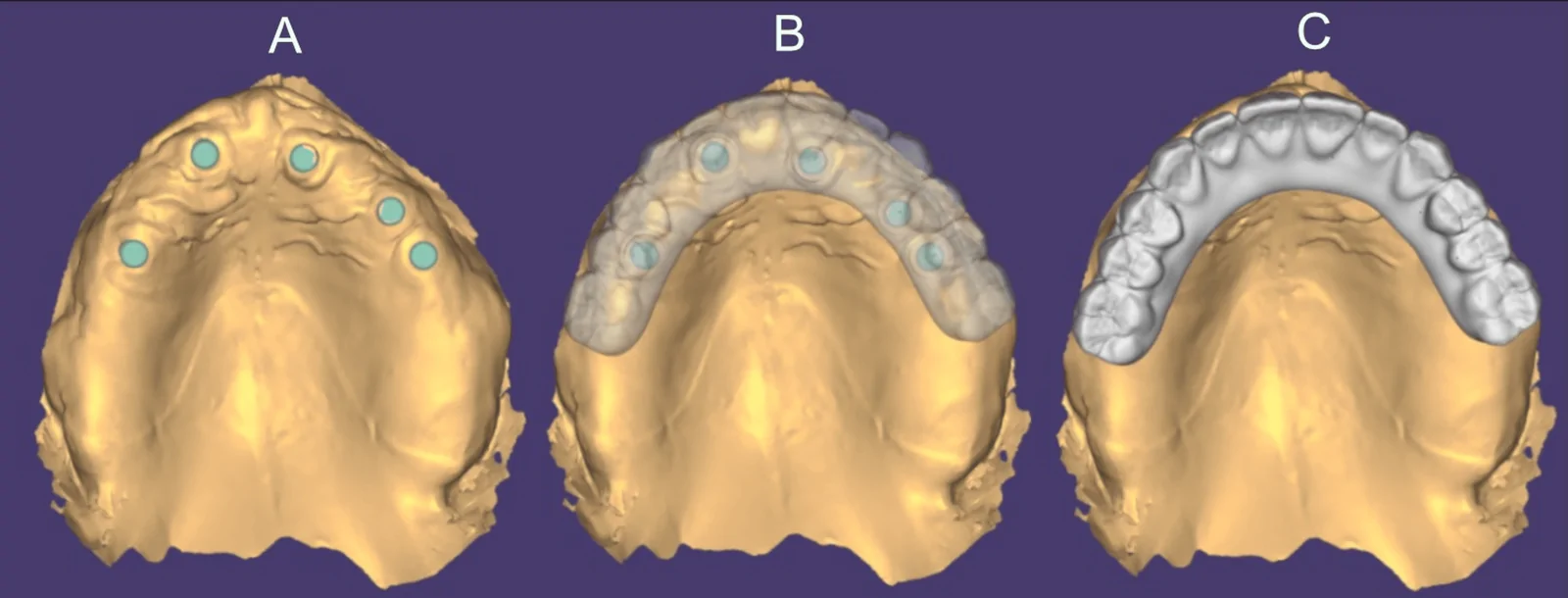
Virtual occlusal views demonstrating the relationship between the implant attachments The virtual occlusal view of the maxillary arch of the LOCATOR® (Zest Dental Solutions, Carlsbad, CA) attachments (A), overdenture related to the attachments (B) and the design of the overdenture (C).

**Figure 13:**
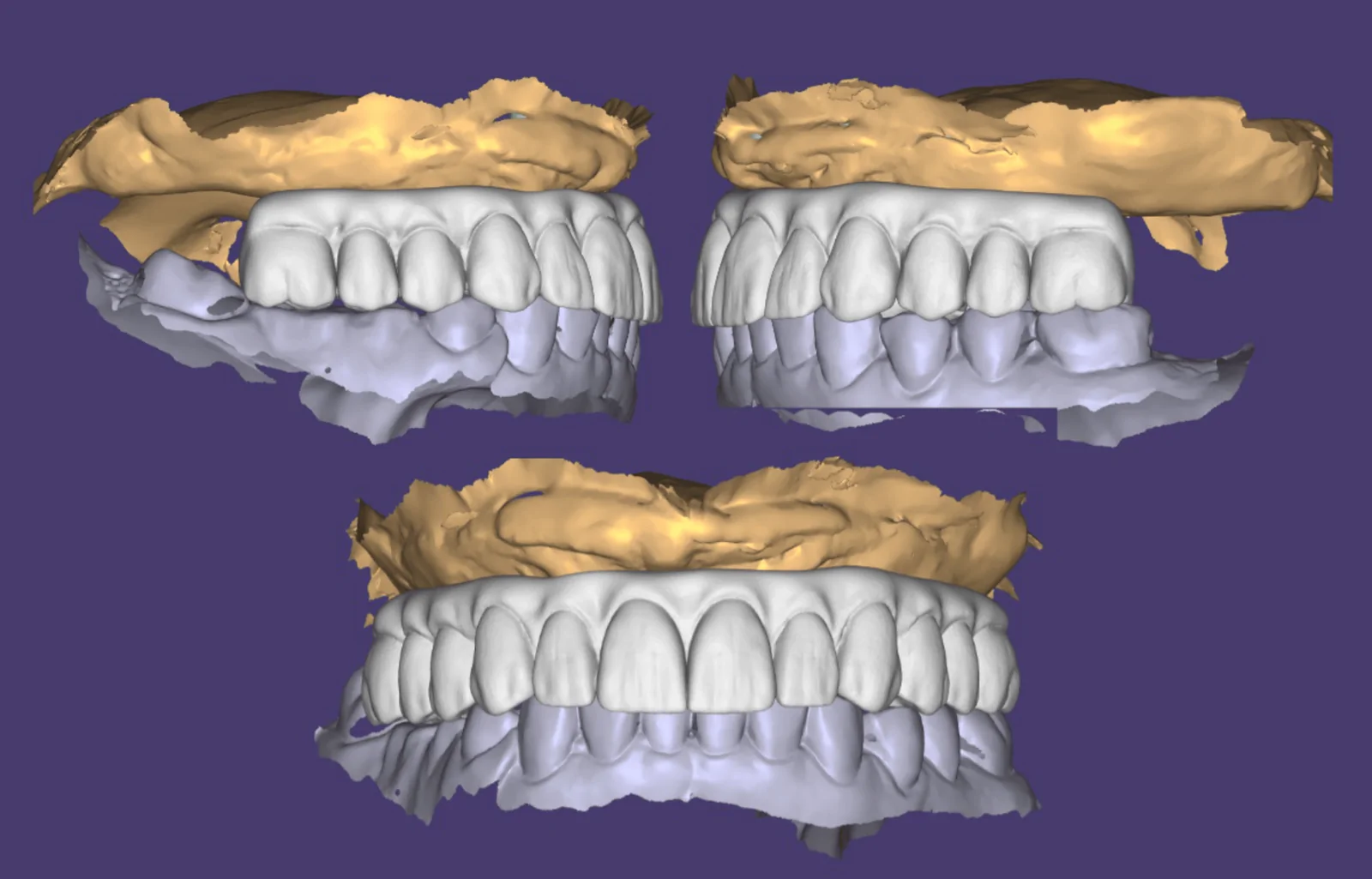
The virtual designed overdenture articulated with the lower arch from various views

**Figure 14:**
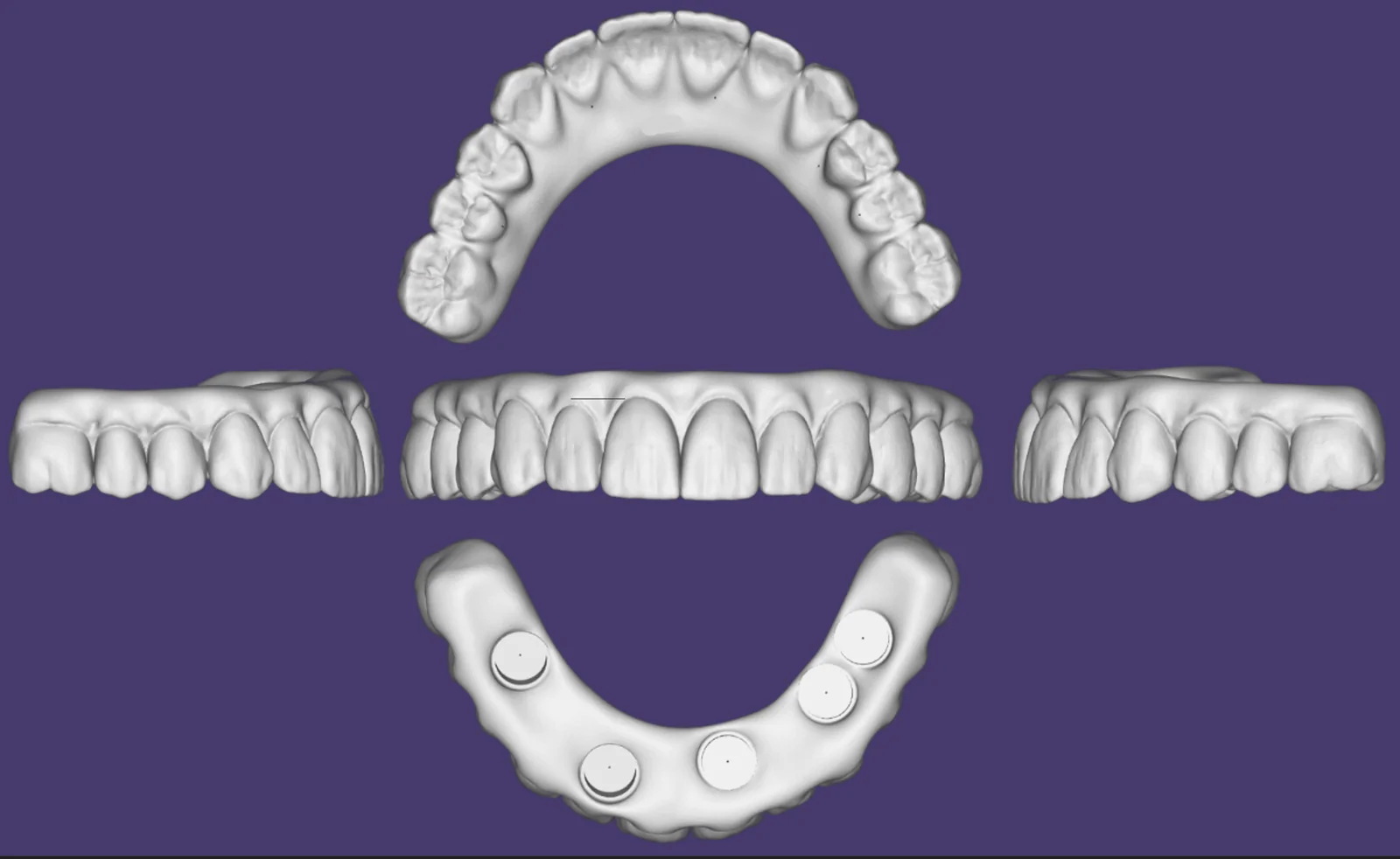
Virtual design of the overdenture from various views

Occlusal views further demonstrate the relationship between the implant attachments, the available restorative space, and the completed overdenture design before fabrication (Figure 12). The completed virtual overdenture is evaluated from multiple perspectives to verify tooth position, contours, occlusion, and attachment relationships before proceeding to manufacturing (Figures 13, 14).

**Figure 15:**
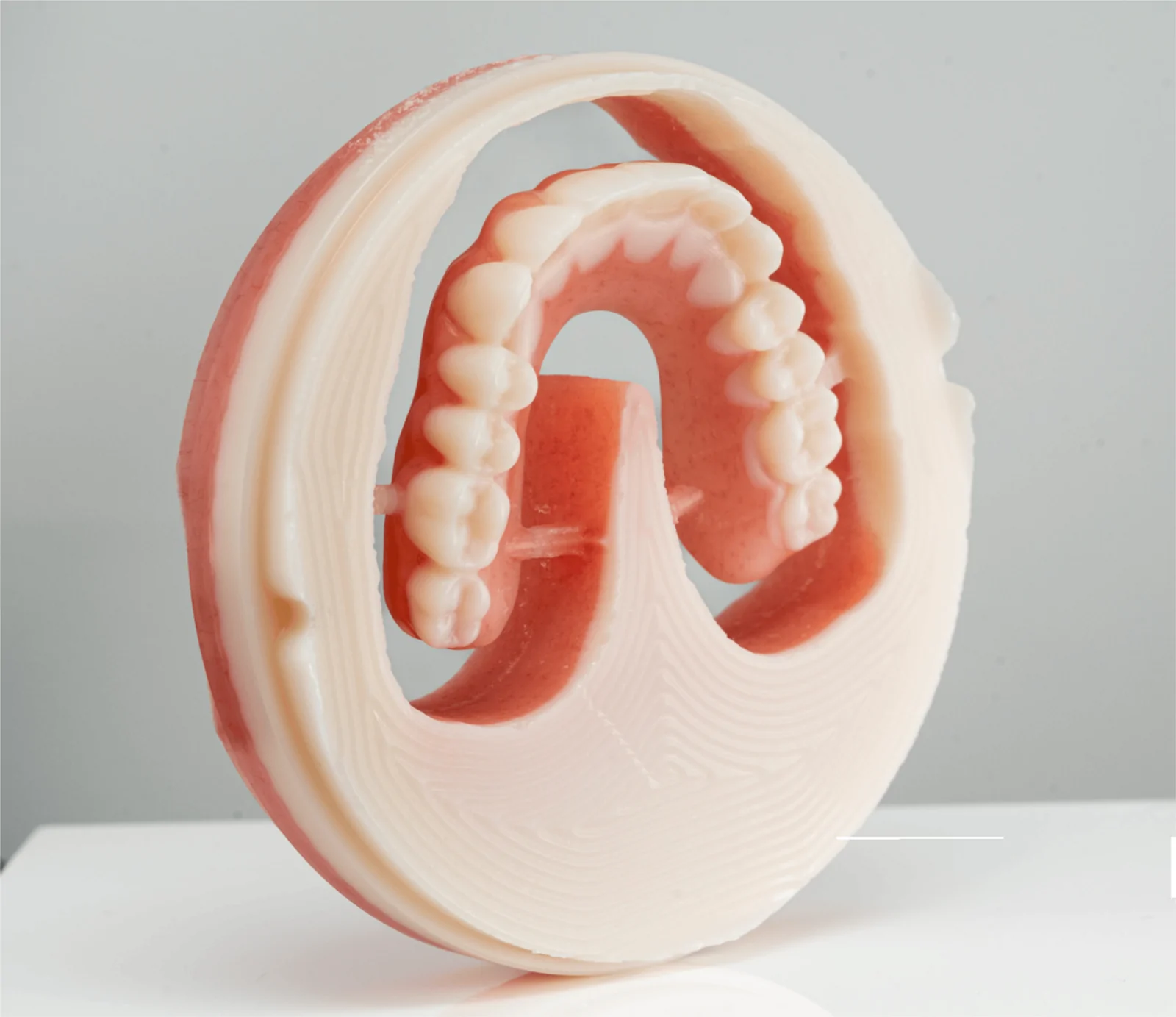
The overdenture following milling from the Ivotion® puck Ivotion® (Ivoclar, FL-9494 Schaan, Principality of Liechtenstein).

**Figure 16:**
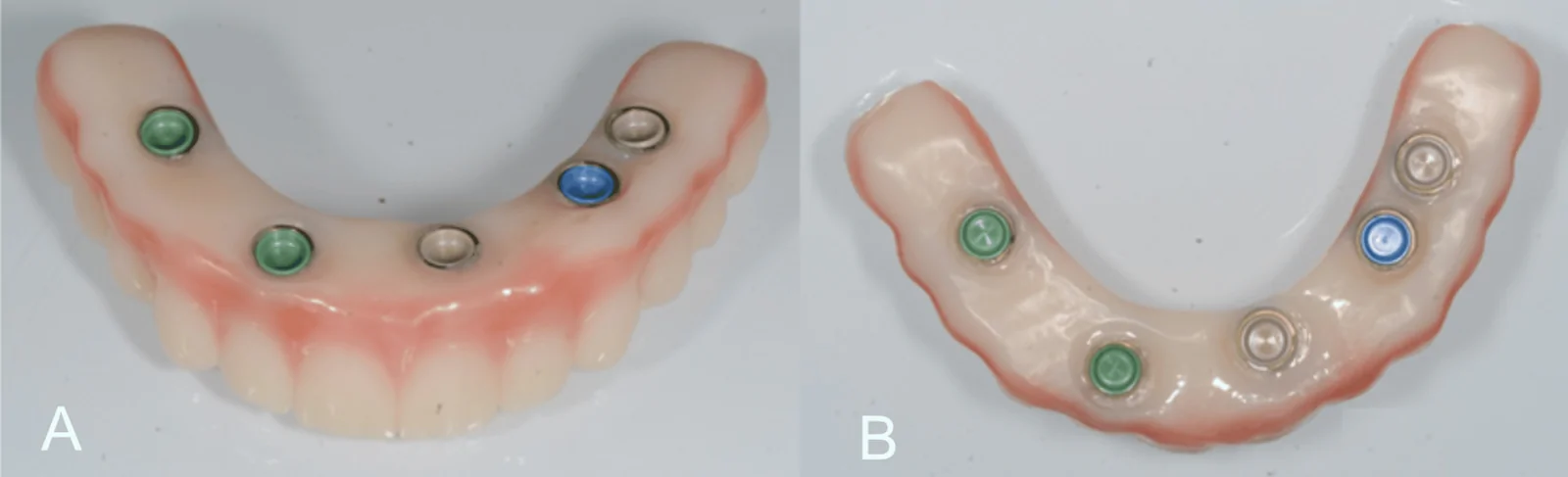
The Ivotion® milled prosthesis with the LOCATOR FIXED® housings incorporated at the laboratory The prosthesis is shown from the facial (A) and intaglio (B) views. LOCATOR FIXED® (Zest Dental Solutions, Carlsbad, CA); Ivotion® (Ivoclar, FL-9494 Schaan, Principality of Liechtenstein).

Milled complete dentures represent another digital treatment option for both conventional complete dentures and implant-supported overdentures. When a monolithic milled prosthesis is selected, the finalized digital design is transferred to the Ivotion® CAD/CAM system (Ivoclar, FL-9494 Schaan, Principality of Liechtenstein), which utilizes an industrially polymerized multilayer polymethyl methacrylate (PMMA) disc containing both denture base and tooth-colored materials in the same disk. In this case, the patient desired a fixed approach rather than a removable overdenture, so the prosthesis would include Locator Fixed attachments instead of Locator removable attachments. This does not change the described prosthesis fabrication other than the Locator attachments used. With the fixed approach, the prosthesis is not removable by the patient but can be removed by the dentist should that be required. Additionally, if needed, the fixed prosthesis can be converted to removable by changing the Locator males in the housings within the prosthesis. The definitive overdenture is milled as a single monolithic restoration (Figure 15), after which the LOCATOR® Fixed metal housings are luted into the prosthesis (Figure 16) at the laboratory utilizing the proprietary patent-pending processing technique. Compared with conventional denture processing, milled dentures provide improved dimensional accuracy and material consistency because they are fabricated from industrially polymerized cross-hatched PMMA blanks. The digital workflow also preserves the design files, allowing efficient fabrication of a replacement prosthesis if required. Although milled dentures generally involve higher laboratory and material costs than printed dentures, they remain a predictable treatment option when a monolithic prosthesis with established CAD/CAM manufacturing characteristics is desired.

**Figure 17:**
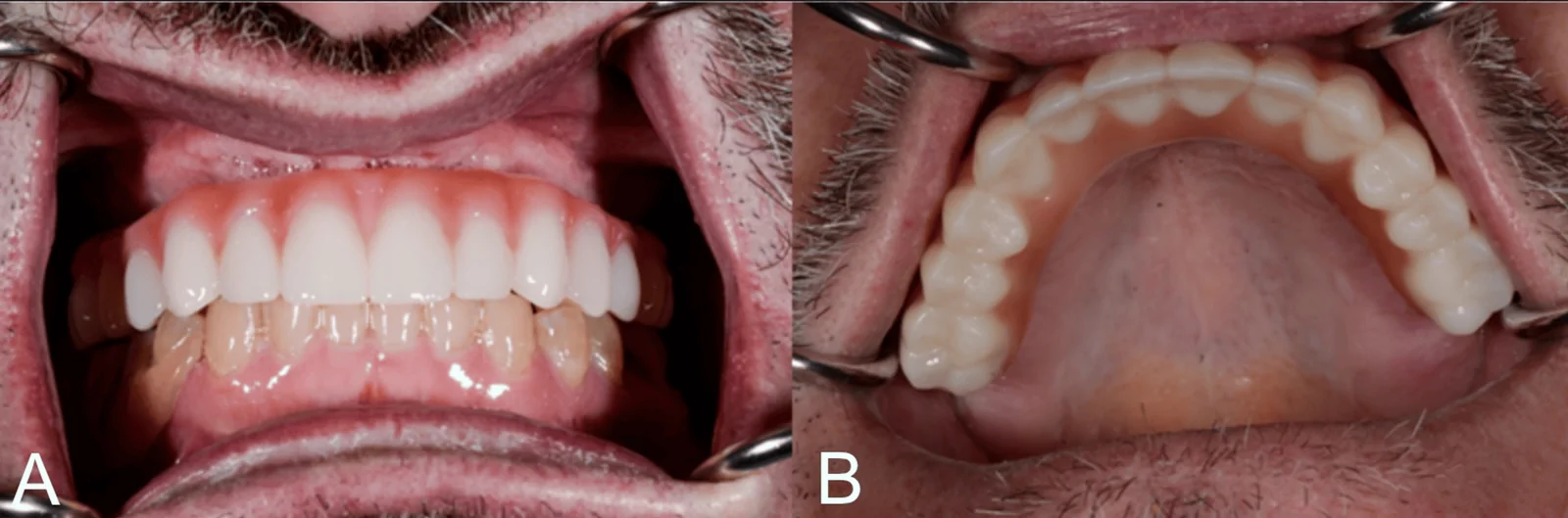
The definitive maxillary LOCATOR FIXED® prosthesis in occlusion (A) Facial and (B) occlusal (B) views. LOCATOR FIXED® (Zest Dental Solutions, Carlsbad, CA).

**Figure 18:**
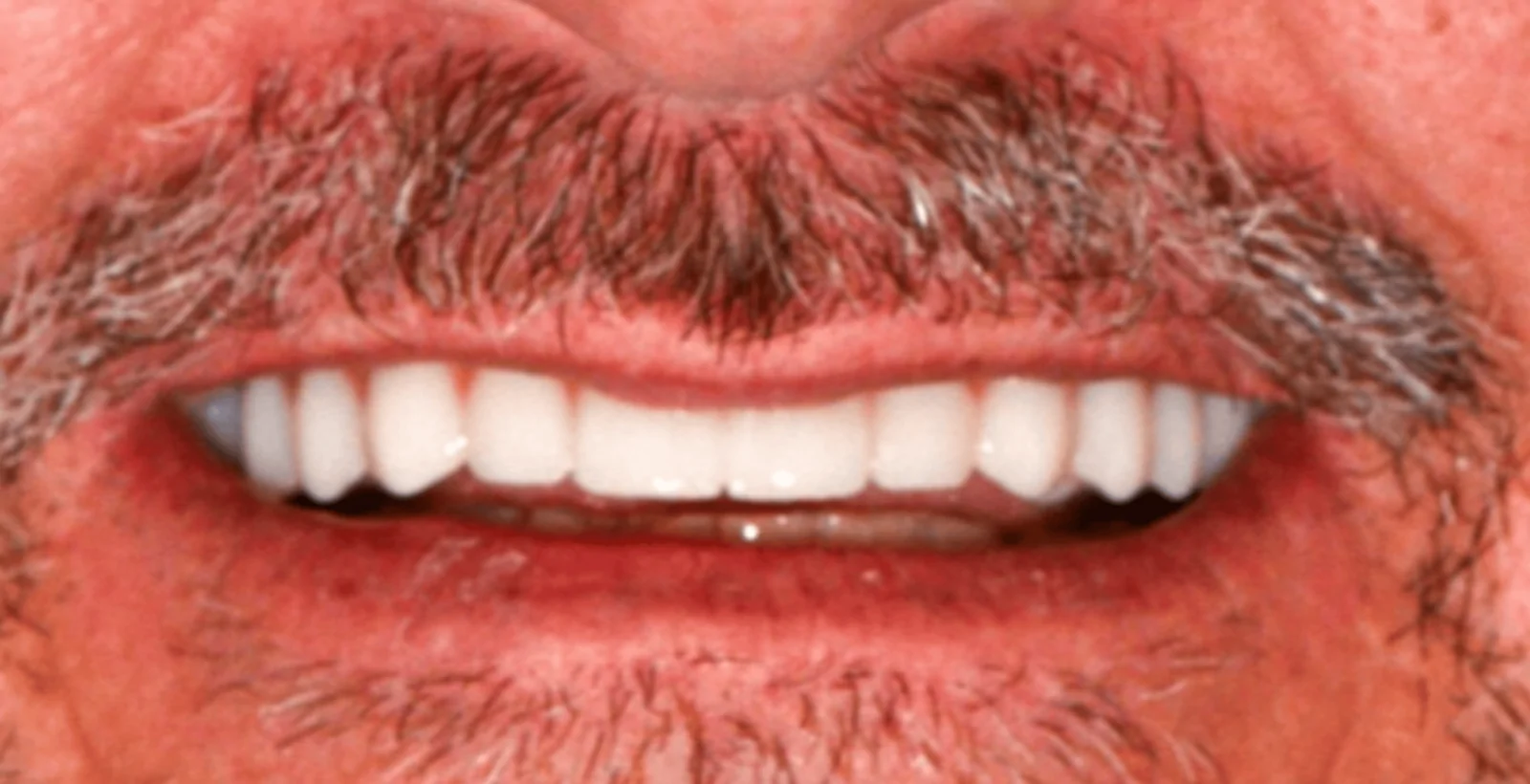
Patient's dental image after delivery of the definitive prosthesis The resulting natural smile following delivery of the definitive maxillary LOCATOR FIXED® (Zest Dental Solutions, Carlsbad, CA) prosthesis.

The patient returned to the practice, and the definitive overdenture is inserted and evaluated intraorally for complete seating, passive fit, occlusion, retention, phonetics, and esthetics (Figure 17). Minor occlusal adjustments are performed as needed before the patient is instructed on hygiene procedures and maintenance of the LOCATOR® Fixed attachment system. The completed restoration restores function and esthetics while maintaining the established VDO, tooth arrangement, occlusal scheme, and facial support developed throughout the digital workflow (Figure 18).

## Discussion

Implant-supported overdentures remain one of the most predictable treatment options for rehabilitating edentulous patients, consistently improving retention, stability, masticatory efficiency, patient satisfaction, and oral health-related quality of life compared with conventional complete dentures [1-4]. Long-term success, however, depends on more than implant survival. Accurate transfer of implant position, preservation of established prosthetic parameters, and efficient long-term maintenance are equally important to predictable clinical outcomes.

Conventional implant impressions using splinted impression copings remain highly accurate and continue to serve as the benchmark for many full-arch restorations [8-10,17]. Their primary disadvantage is not accuracy, but the number of clinical and laboratory procedures required to fabricate and maintain the definitive prosthesis. Conventional workflows often require multiple impressions, master casts, verification jigs, jaw relation records, and laboratory remounting. When an overdenture requires replacement years later, many of these procedures must be repeated if the original records are unavailable.

Digital workflows have simplified many restorative procedures while improving communication between the clinician and laboratory. Numerous investigations have demonstrated excellent accuracy for digital implant impressions, particularly in partially edentulous patients, with continued improvements in scanner technology expanding their application to complete-arch implant therapy [5-16]. Despite these advances, complete-arch scanning remains technique sensitive because the absence of stable anatomic landmarks increases the potential for cumulative stitching errors. Proper scanning protocols and meticulous clinical technique therefore remain essential for predictable results.

The ArchBridge™ for Overdenture workflow addresses these challenges by preserving more than implant position alone. By digitally capturing the patient's existing prosthesis, the workflow also preserves tooth arrangement, VDO, facial support, phonetics, occlusal relationships, and soft tissue contours that have already been clinically validated. Rather than recreating these records during fabrication of the definitive prosthesis, they are transferred directly into the digital workflow, reducing both clinical and laboratory procedures while maintaining restorative accuracy and eliminating the need for any chairside pickup of housings.

The laboratory merges these datasets into a single digital model that reproduces implant position, soft tissue architecture, occlusion, VDO, and prosthetic contours. Using this information, the definitive overdenture can be designed and fabricated with CAD/CAM technology while preserving the esthetics and occlusion that have already been clinically validated. The precision achieved with CAD/CAM-manufactured implant frameworks has been well documented and contributes to improved passive fit and restorative accuracy [18,19].

From a clinical perspective, one of the greatest advantages of this approach is long-term prosthetic maintenance. Implant-supported overdentures predictably require replacement of attachment inserts, relines, repair of fractured denture bases or prosthetic teeth, and, ultimately, fabrication of replacement prostheses during years of service [20-23]. Because implant position and prosthetic design are permanently archived, many of these procedures can often be completed with substantially fewer clinical appointments and without repeating conventional impressions or jaw relation records. This has the potential to reduce chair time, improve laboratory efficiency, and enhance patient convenience throughout the restoration’s life.

The ArchBridge™ approach can also be considered within the broader development of intraoral photogrammetry and enhanced complete-arch implant scanning techniques. Systems using distinctive or interconnected scan-body geometries have been developed to provide more reliable reference points across an edentulous arch and reduce the cumulative registration errors associated with conventional sequential intraoral scanning. Similar concepts have been incorporated into systems such as Scan Ladder and TruAbutment IOConnect. Recent investigations of intraoral photogrammetry and noncalibrated splinting techniques have demonstrated improved complete-arch implant scanning accuracy compared with conventional nonsplinted scan-body techniques. These findings support the underlying concept of using enhanced geometric references to improve full-arch implant registration; however, accuracy demonstrated with one system cannot be directly extrapolated to another. Independent studies are therefore needed to quantitatively determine the trueness and precision of implant-position transfer obtained with the ArchBridge™ workflow.

It is important to recognize that digital technology does not replace sound prosthodontic principles. Accurate seating of overdenture abutments and scan bodies, adherence to recommended scanning protocols, verification of passive framework fit, and careful evaluation of occlusion remain essential regardless of the impression technique used. Digital workflows improve efficiency, but predictable long-term success continues to depend on meticulous diagnosis, treatment planning, and clinical execution.

The ArchBridge™ for Overdenture workflow should also be viewed within the expanding spectrum of digital implant technologies. Conventional impressions, intraoral scanning, and hybrid workflows each have appropriate clinical indications. By combining accurate implant registration and the ability to process at the lab model-free with digital preservation of the patient's existing prosthesis, the ArchBridge™ for Overdenture technique offers a practical alternative for implant-supported overdentures that simplifies restorative procedures while maintaining the accuracy required for predictable long-term outcomes.

Limitations

This report describes the clinical application and technical feasibility of the ArchBridge™ workflow using an illustrative clinical case and does not provide a quantitative comparison with conventional impression techniques, intraoral photogrammetry, or other digital workflows. The accuracy and precision of implant-position transfer, laboratory housing positioning, treatment efficiency, number of clinical appointments, cost, patient outcomes, complication rates, and long-term prosthetic performance have not yet been established. Prospective comparative clinical studies are needed to evaluate these outcomes and determine the relative advantages and limitations of the workflow. Additionally, the laboratory processing of the attachment housings utilizes a proprietary patent-pending technique, and detailed processing parameters were not available to the authors; therefore, this component of the workflow cannot be independently reproduced from the information presented in this report. Additional case-specific information, including detailed patient characteristics, implant positions, abutment heights and torque values, scanner and software versions, and certain manufacturing parameters, was not available for retrospective reporting.

## Conclusions

Implant-supported overdentures continue to provide a predictable and cost-effective solution for the rehabilitation of edentulous patients. The ArchBridge™ for Overdenture digitally integrated, model-free workflow combines implant registration with digital capture of the patient’s existing prosthesis, preserving clinically established parameters including implant position, VDO, occlusion, tooth arrangement, facial support, and soft tissue contours. This information is incorporated into a single digital record that may streamline fabrication of the definitive prosthesis. Although a light-body PVS reline is used to capture the supporting tissues, the subsequent records are incorporated into the digital workflow, eliminating the need for conventional stone models and chairside pickup of the retentive housings. Using a proprietary patent-pending processing technique, the laboratory incorporates the housings directly from the digital records in a model-free workflow, with the potential to reduce chair time, laboratory procedures, and technique-sensitive steps.

An additional potential advantage is the creation of a permanent digital archive containing implant-position and prosthetic design information. These records may facilitate future repair, duplication, or replacement of the prosthesis; however, they do not replace appropriate clinical reassessment. A future reline requires an updated record of the supporting tissues, and conversion to a different prosthetic design requires reassessment of restorative space, tissue contours, implant and peri-implant conditions, occlusion, and hygiene access. The archived digital information should therefore be considered a reference that may simplify future treatment rather than a substitute for updated clinical records when conditions have changed. Although this clinical technique report demonstrates the feasibility of the ArchBridge™ workflow, it does not establish superiority over conventional or other digital workflows. The accuracy and precision of implant-position transfer, housing-positioning accuracy, treatment efficiency, patient outcomes, and long-term prosthetic performance have not yet been established. Prospective comparative clinical studies are needed to quantitatively evaluate these parameters and determine the relative advantages and limitations of the workflow.
